# Automated Tractography of Corticospinal and Corticoreticular Projections Originating From Brodmann Area 44 in Patients With Mild Stroke

**DOI:** 10.7759/cureus.116866

**Published:** 2026-09-24

**Authors:** Tetsuo Koyama, Midori Mochizuki, Yuki Uchiyama

**Affiliations:** 1 Department of Rehabilitation Medicine, Nishinomiya Kyoritsu Neurosurgical Hospital, Nishinomiya, JPN; 2 Department of Rehabilitation Medicine, Hyogo Medical University, Nishinomiya, JPN

**Keywords:** alternative pathway, automated tractography, corticoreticular projection, recovery, upper extremity

## Abstract

Background: Although upper-limb motor recovery after stroke is strongly linked to the integrity of the primary corticospinal tract, alternative descending motor pathways originating from secondary motor-related cortical areas, such as Brodmann area 44 (BA44; Broca's area), may also contribute to functional recovery. This study aimed to establish a practical automated diffusion-tensor tractography protocol to evaluate the microstructural properties of corticospinal and corticoreticular projections (CS-P and CR-P) originating from BA44 in patients with mild stroke who achieved favorable motor outcomes.

Methods: We retrospectively investigated patients with first-ever, unilateral thalamic or putaminal hemorrhagic stroke who achieved complete independence in activities of daily living and a Brunnstrom Recovery Stage of ≥5 in both the upper and lower limbs at discharge. Diffusion-tensor imaging was performed during the second week of hospitalization using a 3.0-T MRI scanner (MAGNETOM Trio; Siemens, Erlangen, Germany). The CS-P and CR-P were reconstructed using an automated tractography pipeline, with the cerebral peduncle and gigantocellular reticular nucleus serving as seed regions, respectively, and BA44 serving as the target region. Interhemispheric differences in fractional anisotropy (FA) values between the lesioned and non-lesioned hemispheres were evaluated using the Wilcoxon signed-rank test.

Results: A total of 14 patients were enrolled. Both the CS-P and CR-P originating from BA44 were successfully reconstructed in all subjects. FA values in the lesioned hemisphere were significantly lower than those in the non-lesioned hemisphere for both the CS-P (median (IQR): 0.484 (0.452-0.514) vs. 0.508 (0.474-0.531), S = 39.5, P = 0.011) and the CR-P (0.442 (0.410-0.462) vs. 0.463 (0.446-0.474), S = 31.5, P = 0.049).

Conclusions: Automated diffusion-tensor tractography can reliably detect microstructural alterations in alternative descending motor pathways originating from BA44, even in patients with mild stroke and favorable motor outcomes. This neuroimaging protocol offers a practical and clinically viable approach for assessing neural reorganization in stroke rehabilitation.

## Introduction

Stroke is a major cause of disability worldwide [1]. It frequently affects both motor and cognitive functions [2,3]. To facilitate recovery, rehabilitation programs are commonly implemented [4]. Among the various neurological impairments caused by stroke, upper-limb dysfunction has received significant attention because it is a major source of post-stroke disability [5].

Upper-limb impairment after stroke is strongly associated with damage to the corticospinal tract, a major descending motor pathway originating from the primary motor cortex and other motor-related cortical areas [6]. However, some degree of motor recovery may occur even after substantial damage to the corticospinal system [7]. Neuroanatomical studies indicate that the premotor and supplementary motor cortices also give rise to corticospinal projections, which may provide alternative descending routes contributing to motor recovery [8]. Another possibility is the recruitment of the corticoreticular pathway, which may contribute to motor recovery through the reticulospinal system, particularly in the control of proximal and gross upper-limb movements [9,10].

The aim of this study was to establish a diffusion-tensor tractography methodology for assessing potential neural substrates of upper-extremity motor control in patients with stroke. Among the motor-related cortical regions outside the primary motor cortex, Brodmann area 44 (BA44), also known as Broca's area, may contribute to upper-limb motor representations [11,12]. This study focused on the corticospinal and corticoreticular projections (CS-P and CR-P, respectively) identified by tractography using BA44 as a cortical target region.

## Materials and methods

Patients

This study extends our previous work investigating the clinical applicability of automated tractography in patients with stroke [13-15]. This retrospective observational study utilized a retrospective review of medical records at Nishinomiya Kyoritsu Neurosurgical Hospital. We identified patients who were hospitalized for stroke at Nishinomiya Kyoritsu Neurosurgical Hospital between April 2021 and March 2024. To minimize potential confounding due to baseline functional heterogeneity and lesion variability, selection was restricted to individuals experiencing their first-ever, unilateral thalamic or putaminal hemorrhagic stroke who had been independent in activities of daily living before the current hospitalization [13-15]. Patients with pre-existing neurodegenerative disorders, such as Alzheimer's or Parkinson's disease, were excluded. Furthermore, patients who experienced an acute disturbance of consciousness during their clinical course or had severe concurrent systemic comorbidities were excluded. The inclusion and exclusion criteria are summarized in Table 1.

**Table 1:** Inclusion and exclusion criteria for the study population.

Category	Criteria
Inclusion criteria	Patients after first-ever thalamic or putaminal hemorrhagic stroke
	Independent in activities of daily living before admission
	Admitted to Nishinomiya Kyoritsu Neurosurgical Hospital
	Discharged home directly from our acute care unit
Exclusion criteria	Pre-existing neurological disorders (e.g., Alzheimer’s or Parkinson’s disease)
	Pronounced neurological deterioration during the acute phase
	Severe medical complications during the acute phase

Because the core objective of this study was to refine the clinical utility of automated tractography [13-15], sample selection was tailored to facilitate precise interpretation of microstructural findings. Specifically, we enrolled individuals who achieved complete independence in daily functioning and were discharged directly home from our acute care unit. Full independence in activities of daily living was defined as a score of 2 or less on the modified Rankin Scale [16], which is widely used to evaluate global disability and functional outcome after stroke. Eligible subjects demonstrated favorable motor outcomes, defined as a Brunnstrom Recovery Stage of 5 or higher in both the affected upper and lower limbs [17]. This standardized scale evaluates motor performance of the extremities across six sequential stages, ranging from flaccidity to near-complete functional independence [17]. Acute cerebral hemorrhage was confirmed by computed tomography performed immediately after transfer to our emergency department [18-20]. All subjects underwent standardized acute-phase medical management in accordance with the Japan Stroke Society Guideline 2021 for the Treatment of Stroke [4]. Throughout their hospitalization, participants underwent structured rehabilitation programs encompassing physical, occupational, and speech therapy, for up to the statutory daily limit of 180 minutes in accordance with Japanese medical insurance regulations [4]. The length of hospital stay (LOS) was recorded upon discharge from our acute care unit. The institutional review board of Hyogo Medical University approved this study (Approval No. 4666), and informed consent was obtained using an opt-out framework.

Diffusion-tensor imaging acquisition

In accordance with our established protocols [13-15], diffusion-tensor imaging examinations were performed during the second week of hospitalization. Scans were obtained using a 3.0-T MRI scanner (MAGNETOM Trio; Siemens, Erlangen, Germany) equipped with a dedicated 32-channel head receiver coil. A single-shot echo-planar imaging sequence was used to acquire diffusion-weighted images in 30 non-collinear encoding directions (b = 1500 s/mm^2^), supplemented by a baseline acquisition without diffusion-weighting (b = 0 s/mm^2^). Whole-brain coverage was achieved using 80 contiguous axial slices, with a 256 × 256 mm field of view, a 128 × 128 acquisition matrix, and a 2-mm slice thickness. Scan parameters included an echo time of 96 ms, a repetition time of 10,900 ms, and a 90° flip angle. To mitigate eddy-current artifacts and susceptibility-related geometric distortions, additional b = 0 s/mm² volumes were acquired using reversed phase-encoding polarities. High-resolution structural reference images were acquired simultaneously using a 3D T1-weighted fast gradient-echo sequence with 176 sagittal slices, a 256 × 256 mm field of view, a 256 × 256 matrix, a 1-mm slice thickness, an echo time of 2.52 ms, a repetition time of 1,900 ms, and a 10° flip angle.

Image processing

The data preprocessing pipeline followed the procedures described in our previous publications [13-15]. Briefly, preprocessing included the removal of Gibbs ringing artifacts, correction for eddy-current effects and echo-planar imaging distortions, and intensity inhomogeneity correction using MRtrix3 [21] and the FMRIB Software Library (FSL) [22]. Individual brain-extraction masks were generated from the bias-corrected datasets and used to constrain subsequent spatial processing. Further procedural parameters and methodological specifications have been described in our previous reports [13-15].

**Figure 1:**
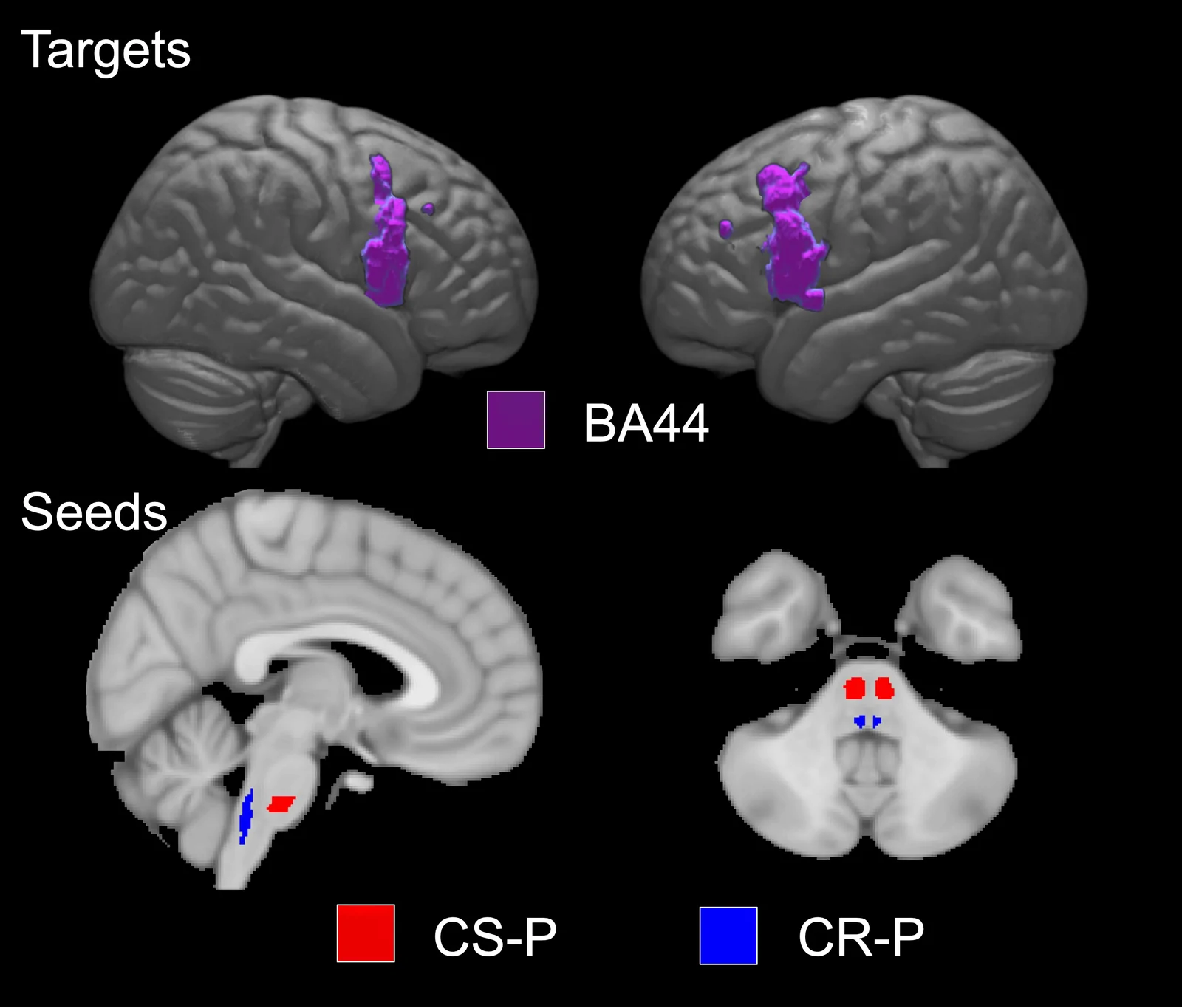
Seed and target regions for the CS-P and CR-P. BA44: Brodmann area 44; CR-P: corticoreticular projection; CS-P: corticospinal projection.

Tractography was performed using FSL's built-in automated command “probtrackx2_gpu.” The primary aim of the present study was to reconstruct the CS-P and CR-P in relation to BA44. The same seed voxels used in our previous study were employed for the CS-P and CR-P (Figure 1) [15]. For the CS-P, the seed mask was placed in the cerebral peduncle [15,23], whereas for the CR-P, the seed mask was placed in the gigantocellular reticular nucleus (Figure 1) [15,24]. As in our previous study, streamline generation criteria included the exclusion masks and the number of samples (“--nsamples=3000” option) used for corticospinal tract tracking in the “XTRACT” pipeline implemented in FSL [23]. The target region for fiber tracking was BA44, defined using the Julich-Brain atlas (left hemisphere index 13 and right hemisphere index 14) implemented in FSL [25]. Because fractional anisotropy (FA) indices are known to vary according to the chosen filtering threshold, we adopted a uniform cutoff value of 0.01 in accordance with our established methodology [13-15]. Both tract volume and mean FA were subsequently calculated for each pathway. Due to their anatomical proximity, partial spatial overlap between the CS-P and CR-P was considerable [15]. To account for this anatomical overlap, the volumes of the intersecting regions were measured separately. All imaging data were analyzed using a built-to-order, specialized workstation (POWERSTEP Tower for Lin4Neuro; Amulet Inc., Tokyo, Japan) equipped with the Lin4Neuro software platform [26].

Statistical analysis

Because our patient series consisted of individuals with mild neurological deficits, interhemispheric differences in FA values between the lesioned and non-lesioned hemispheres were evaluated using the Wilcoxon signed-rank test in JMP software (version 19; SAS Institute, Cary, North Carolina). A P value of <0.05 was considered statistically significant. Prior to data analysis, our analytical plan was pre-specified to focus inferential testing exclusively on FA values. Although tract volumes were also measured, they were presented descriptively rather than used for inferential comparisons, because our previous studies demonstrated that tract volumes do not reliably reflect clinical symptoms or motor outcomes in this patient population [13-15]. Given the exploratory and focused nature of this preliminary study and its small sample size, adjustments for multiple comparisons were not applied, and the findings should be interpreted with caution.

## Results

During the study period, a total of 85 patients with a first-ever, unilateral thalamic or putaminal hemorrhagic stroke who had been independent in activities of daily living before admission were identified. Of these, 71 patients were excluded because they were transferred to convalescent rehabilitation facilities (70 patients) or discharged to a nursing home (one patient) in accordance with the Japanese healthcare system. Consequently, the remaining 14 patients who fulfilled all inclusion criteria, including direct discharge home from our acute care unit and a Brunnstrom Recovery Stage of 5 or higher in both the upper and lower limbs, were ultimately analyzed.

Table 2 summarizes the demographic and clinical characteristics of the 14 enrolled patients (eight females and six males; median age, 63 years; range, 51-79 years). The lesions were located in the left hemisphere in eight patients and in the right hemisphere in six, involving the putamen in 10 patients, the thalamus in three patients, and both the putamen and thalamus in one patient. For each patient, a single diffusion-tensor assessment was performed during the second week of hospitalization (range, 8-13 days after admission). The median LOS was 13 days (range, 9-42 days).

**Table 2:** Patient characteristics. BRS scores are presented in the order of proximal upper extremity, distal upper extremity, and lower extremity. BRS: Brunnstrom Recovery Stage; LOS: length of hospital stay.

No.	Age (years)	Sex	Lesioned hemisphere	Lesion site	Timing of imaging (day after admission)	LOS (days)	BRS
1	63	Female	Left	Putamen	9	12	6-6-6
2	67	Male	Right	Thalamus	8	13	6-6-6
3	63	Male	Left	Putamen	8	16	6-6-6
4	65	Male	Right	Thalamus	10	24	6-6-6
5	73	Male	Left	Putamen	8	10	6-6-6
6	66	Female	Left	Putamen	8	9	6-6-6
7	58	Female	Right	Putamen	13	15	6-5-6
8	63	Female	Right	Putamen	12	14	6-6-6
9	63	Female	Left	Putamen	9	16	6-6-6
10	58	Female	Left	Putamen/Thalamus	10	13	6-6-6
11	52	Female	Left	Putamen	10	11	6-6-6
12	79	Female	Right	Thalamus	8	42	6-5-6
13	51	Male	Left	Putamen	8	11	6-6-6
14	55	Male	Right	Putamen	10	13	6-5-6

**Figure 2:**
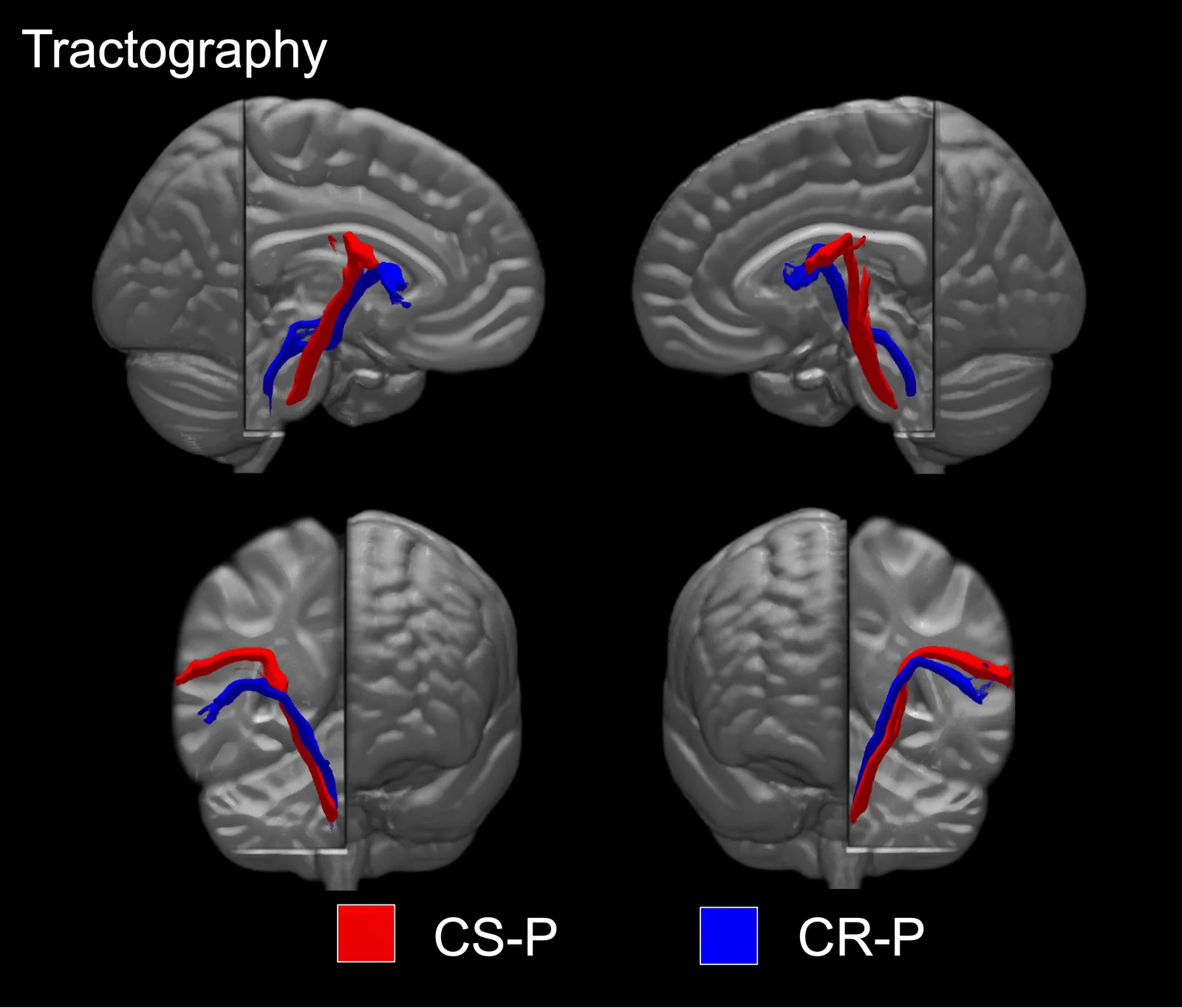
Tractography from an example case. An example (Patient 8 in Tables 2, 3) of the CS-P and CR-P reconstructions. CR-P: corticoreticular projection; CS-P: corticospinal projection.

Table 3 summarizes the quantitative diffusion metrics for all 14 patients, and Figure 2 presents representative CS-P and CR-P reconstructions from Patient 8. The automated protocol successfully reconstructed both pathways in both the lesioned and non-lesioned hemispheres in all subjects.

**Table 3:** Results obtained from automated tractography. CR-P: corticoreticular projection; CS-P: corticospinal projection; FA: fractional anisotropy.

Hemisphere	Lesioned hemisphere					Non-lesioned hemisphere				
Metric	Volume (mL)			FA		Volume (mL)			FA	
No.	CS-P	CR-P	Overlap	CS-P	CR-P	CS-P	CR-P	Overlap	CS-P	CR-P
1	2.45	2.19	0.18	0.516	0.490	1.82	3.20	0.68	0.530	0.463
2	2.46	3.71	0.29	0.427	0.396	1.32	1.39	0.00	0.474	0.450
3	3.49	4.06	1.10	0.489	0.470	3.24	2.52	1.01	0.507	0.463
4	2.94	2.68	0.74	0.527	0.495	2.84	3.53	1.21	0.552	0.476
5	1.38	3.10	0.29	0.479	0.429	3.67	3.09	0.30	0.470	0.487
6	3.42	3.40	1.45	0.423	0.438	2.06	3.02	0.78	0.489	0.473
7	3.95	6.28	1.39	0.467	0.354	3.15	3.02	0.65	0.527	0.470
8	2.58	2.93	0.27	0.513	0.457	1.97	1.22	0.10	0.546	0.516
9	3.42	2.88	0.81	0.452	0.411	2.30	3.41	1.18	0.474	0.446
10	4.65	4.94	1.27	0.460	0.459	4.58	2.48	0.12	0.464	0.446
11	2.02	3.55	0.78	0.489	0.408	2.62	2.90	0.33	0.508	0.441
12	4.07	3.85	1.23	0.513	0.452	2.69	2.56	0.36	0.518	0.447
13	2.42	3.73	0.78	0.542	0.441	2.98	2.06	0.93	0.507	0.467
14	4.65	3.69	0.93	0.452	0.443	2.32	3.66	0.59	0.534	0.445

In the lesioned hemisphere, the median volumes of the CS-P and CR-P were 3.18 mL (range, 1.38-4.65 mL) and 3.62 mL (range, 2.19-6.28 mL), respectively, with a median spatial overlap volume of 0.80 mL (range, 0.18-1.45 mL). The corresponding median FA values in the lesioned hemisphere were 0.484 (range, 0.423-0.542) for the CS-P and 0.442 (range, 0.354-0.495) for the CR-P. In the non-lesioned hemisphere, the median volumes of the CS-P and CR-P were 2.66 mL (range, 1.32-4.58 mL) and 2.96 mL (range, 1.22-3.66 mL), respectively, with a median spatial overlap volume of 0.62 mL (range, 0.00-1.21 mL). The corresponding median FA values in the non-lesioned hemisphere were 0.508 (range, 0.464-0.552) for the CS-P and 0.463 (range, 0.441-0.516) for the CR-P.

Table 4 presents the interhemispheric comparisons of FA values. FA values in the lesioned hemisphere were significantly lower than those in the non-lesioned hemisphere for both the CS-P (median, 0.484; IQR, 0.452-0.514 vs. median, 0.508; IQR, 0.474-0.531; S = 39.5, P = 0.011) and the CR-P (median, 0.442; IQR, 0.410-0.462 vs. median, 0.463; IQR, 0.446-0.474; S = 31.5, P = 0.049).

**Table 4:** Results from statistical comparisons. Data are presented as median (IQR). Interhemispheric differences in FA values between the lesioned and non-lesioned hemispheres were evaluated using the Wilcoxon signed-rank test, and a P-value of <0.05 was considered statistically significant. CR-P: corticoreticular projection; CS-P: corticospinal projection; FA: fractional anisotropy.

Tract	Lesioned hemisphere FA	Non-lesioned hemisphere FA	S	P-value
CS-P	0.484 (0.452-0.514)	0.508 (0.474-0.531)	39.5	0.011
CR-P	0.442 (0.410-0.462)	0.463 (0.446-0.474)	31.5	0.049

## Discussion

In this study, we investigated the microstructural properties of the CS-P and CR-P originating from BA44 (Broca's area) using an automated tractography pipeline in patients with mild stroke who achieved favorable motor outcomes. Our quantitative evaluation demonstrated that both the CS-P and CR-P arising from BA44 could be successfully reconstructed in all enrolled subjects. Notably, FA values in these pathways were significantly lower in the lesioned hemisphere than in the non-lesioned hemisphere (CS-P: P = 0.011; CR-P: P = 0.049), despite the patients exhibiting favorable motor outcomes (Brunnstrom Recovery Stage ≥ 5). To the best of our knowledge, this is the first study to quantitatively demonstrate microstructural alterations in BA44-originating descending motor tracts in patients with well-recovered upper-limb function following subcortical stroke.

In this study, we specifically focused on BA44 as a cortical target region rather than BA45. While Broca's area classically comprises both BA44 and BA45 [27], BA45 has been more traditionally implicated in semantic and higher-order linguistic processing [28], whereas BA44 is well recognized for its involvement in motor-related functions, including motor preparation and action observation [29], as well as hand-object interactions [30]. The primary purpose of this study was to establish a practical diffusion-tensor tractography protocol for assessing alternative motor pathways, namely, the CS-P and CR-P, originating from motor-related cortices outside the primary motor cortex. Consequently, BA44 was selected as the cortical target region as an anatomical anchor for investigating upper-limb motor recovery and compensatory neural reorganization after stroke.

One of the purposes of this study was to establish a practical protocol for neuroimaging studies in rehabilitation medicine, focusing specifically on the compensatory role of alternative pathways, such as the CS-P and CR-P, originating from motor-related cortices outside the primary motor cortex. To keep the analytical pipeline sufficiently simple for daily clinical practice, we employed straightforward procedures. FSL is one of the most widely used tools in neuroimaging research and includes a variety of standard brain maps [22], such as the Julich-Brain atlas utilized in the present study [25]. Segmentation of BA44 can be performed using basic command-line tools such as “fslmaths” [22]. Although further validation is required, such streamlined and automated techniques may eventually help bridge the gap between advanced neuroimaging and routine clinical workflows, thereby offering a supportive reference for rehabilitation practice [13-15].

In this study, we evaluated FA values rather than tract volume data for the statistical comparisons between the lesioned and non-lesioned hemispheres. In our previous series of automated tractography studies, we routinely assessed both tract volumes and FA values between hemispheres [13-15]. However, tract volume results did not reliably reflect clinical symptoms [13-15]. Accordingly, tract volume data were excluded from the statistical comparisons in the present study.

Several methodological limitations should be acknowledged when interpreting these findings. First, FA values are inherently sensitive to the threshold applied during processing. Although a cutoff value of 0.01 was used in accordance with our established methodology [13-15], no universally standardized threshold has been established, and alternative cutoff values may yield different FA values across scanning and analytical settings. Second, the patient cohort was restricted to individuals with first-ever, unilateral supratentorial hemorrhagic stroke. Although this strict inclusion criterion minimized potential confounding and facilitated interpretation of the imaging findings, it limits the generalizability of the present findings to broader stroke populations, including those with infratentorial lesions. Third, the sample size was small (n = 14), reflecting the single-center, retrospective design and strict case-selection criteria. Furthermore, this study lacked a healthy control group; therefore, the non-lesioned hemisphere served as an internal reference for within-subject comparison rather than a normative baseline, and interhemispheric asymmetry should be interpreted with due caution. Fourth, no manual tractography benchmarking was performed. Although manual tractography is susceptible to operator-dependent variability, comparison with an independent tractography approach could have provided additional validation of the automated method [13-15]. Lastly, regarding the anatomical proximity and substantial spatial overlap between the CS-P and CR-P [15], probabilistic tractography inherently carries potential partial-volume or cross-talk effects on pathway-specific FA measurements; however, our standardized seeding and target-based pipeline successfully delineated both pathways in all cases. Nevertheless, the consistent technical success of CS-P and CR-P reconstruction involving BA44 across all cases supports the practical feasibility of the proposed approach. Despite these limitations, the automated tractography framework presented here may provide a useful foundation for future studies investigating alternative motor pathways in stroke rehabilitation.

## Conclusions

Using an automated tractography pipeline with BA44 as a cortical target region, we successfully reconstructed the CS-P and CR-P in patients with mild stroke who achieved favorable motor outcomes. Interhemispheric comparisons revealed significantly lower FA values in the lesioned hemisphere than in the non-lesioned hemisphere for both tracts. Although these cross-sectional observations do not directly establish longitudinal neural reorganization or causal links to functional recovery, these findings demonstrate that automated diffusion-tensor tractography can reliably detect microstructural alterations in alternative descending motor pathways originating from BA44, offering a practical neuroimaging protocol for clinical rehabilitation.
